# Supplementary material for: Outcomes after Flow Diverter Treatment in Subarachnoid Hemorrhage: A Meta-Analysis and Development of a Clinical Prediction Model (OUTFLOW)
Source: Brain Sci. 2022 Mar 15;12(3):394. doi: 10.3390/brainsci12030394 (PMC8946659; doi:10.3390/brainsci12030394)
Supplement: Supplementary file 1 [file brainsci-12-00394-s001.zip › brainsci-1608530-supplementary.pdf]

SUPPLEMENTARY MATERIAL CONTENT

Figure S1: Prisma flow diagram.

Figure S2: Funnel plot.

Table S1: Study characteristics and baseline characteristics per study.

Table S2: Types and number of used flow diverters

Table S3: Outcomes per study.

Table S4: Complications per study.

Table S5: Quality of evidence.

FIGURE S1: PRISMA FLOW DIAGRAM

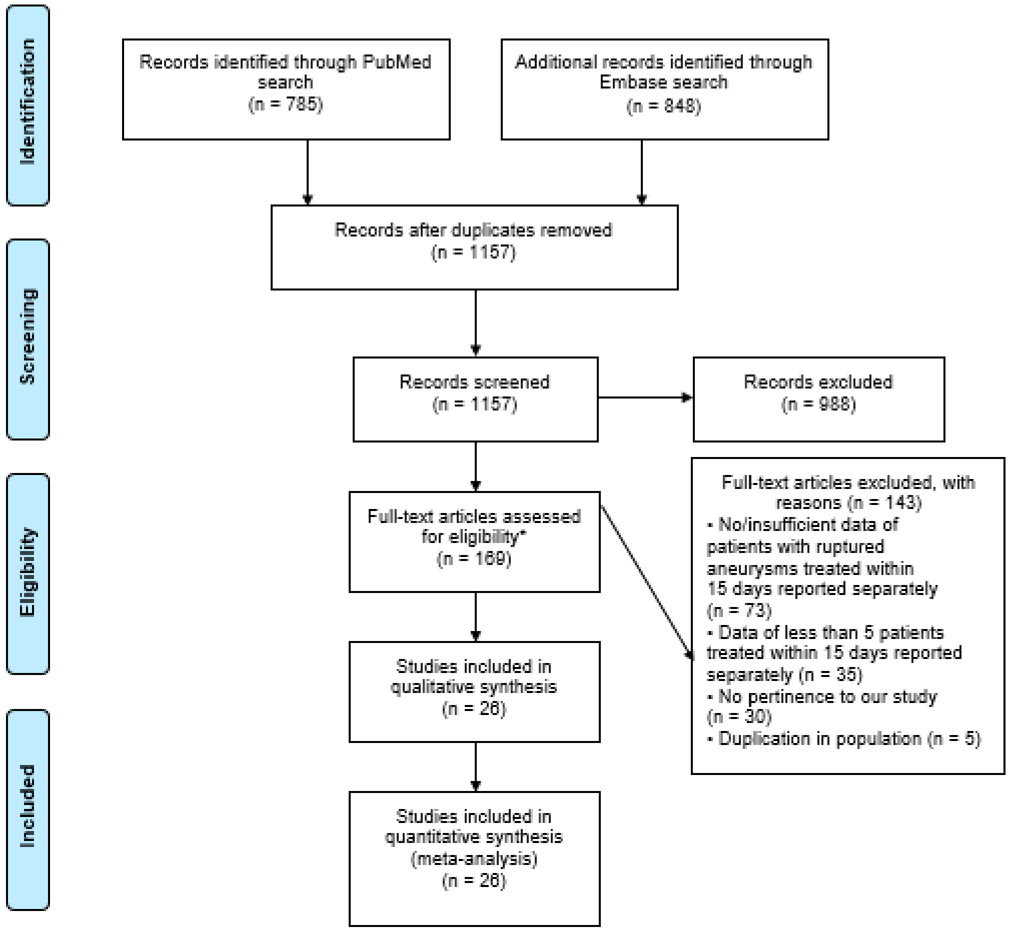

Figure legend (Figure S1):

Inclusion criteria:

- Articles reporting treatment of patients with recently ruptured aneurysms with flow diverters
- Maximum treatment delay after last moment of hemorrhage is $\leq$ 15 days
- N included patients is $\geq$5
- Any type of flow diverter is included
- Also FD additional coiling
- Study must report both clinical (e.g., mRS, GOS) AND radiological outcome (e.g., RR, OKM)
- Studies must report complications;

Exclusion criteria

- Congress abstract
- Posters
- Review
- Reply/commentary
- Studies published in language other than English
- Animal/in vitro studies

FIGURE S2: BIAS FUNNEL PLOTS.

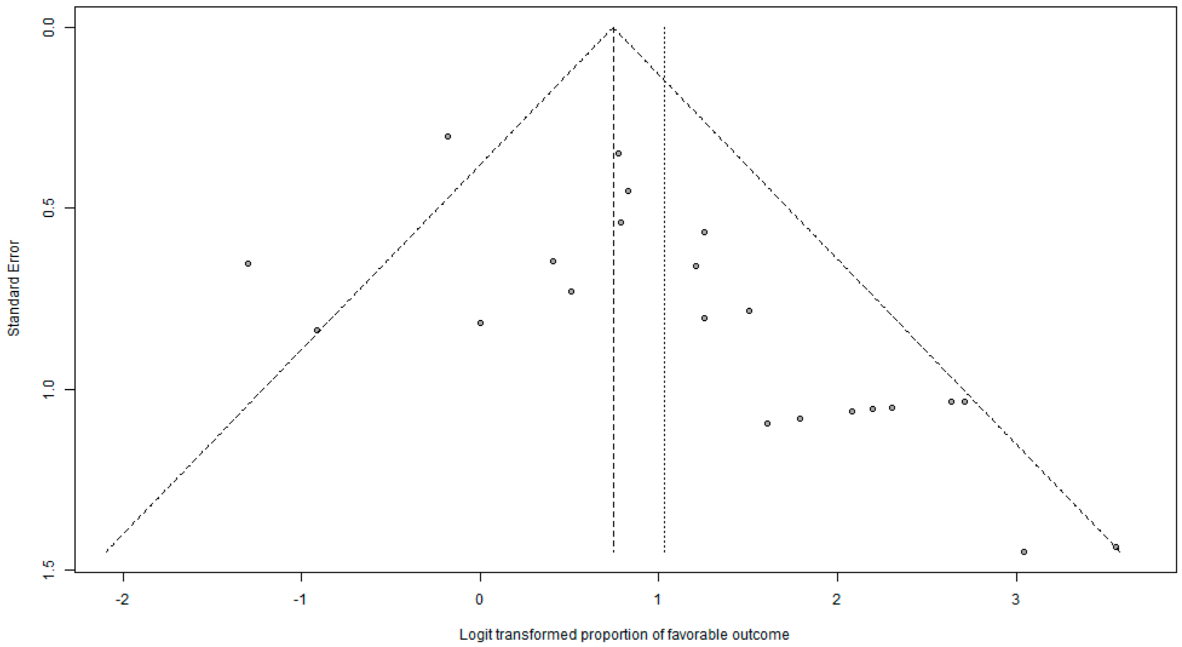

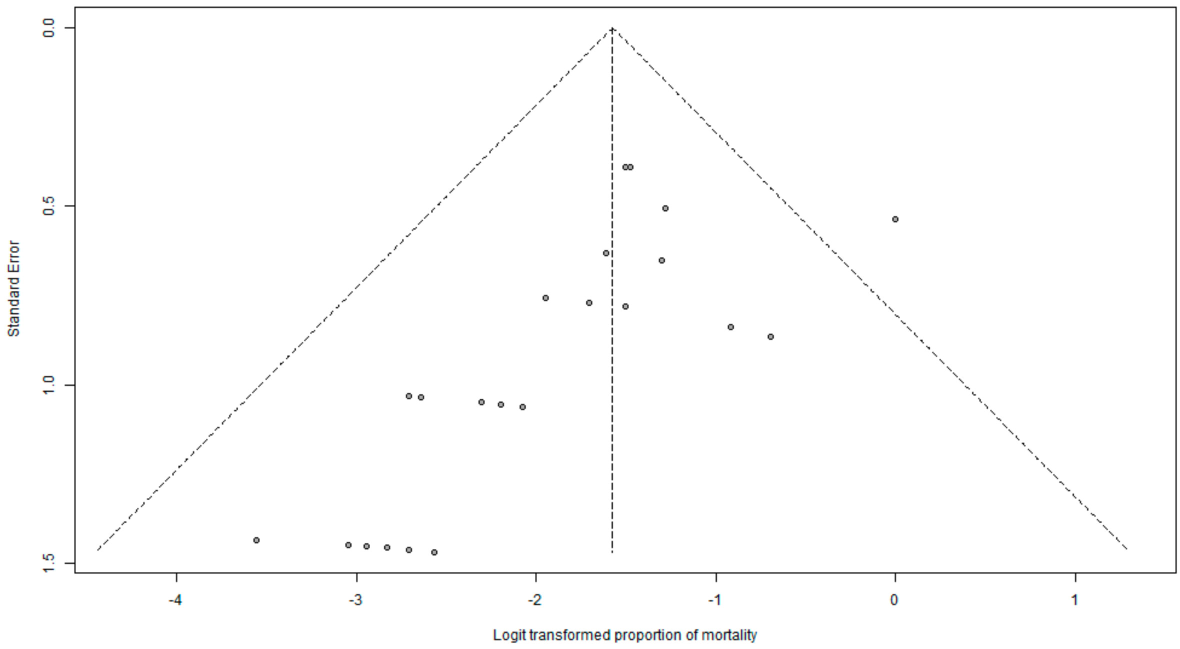
0.0
0.5
1.0
1.5
Standard Error
-4
-3
-2
-1
0
1
Logit transformed proportion of mortality

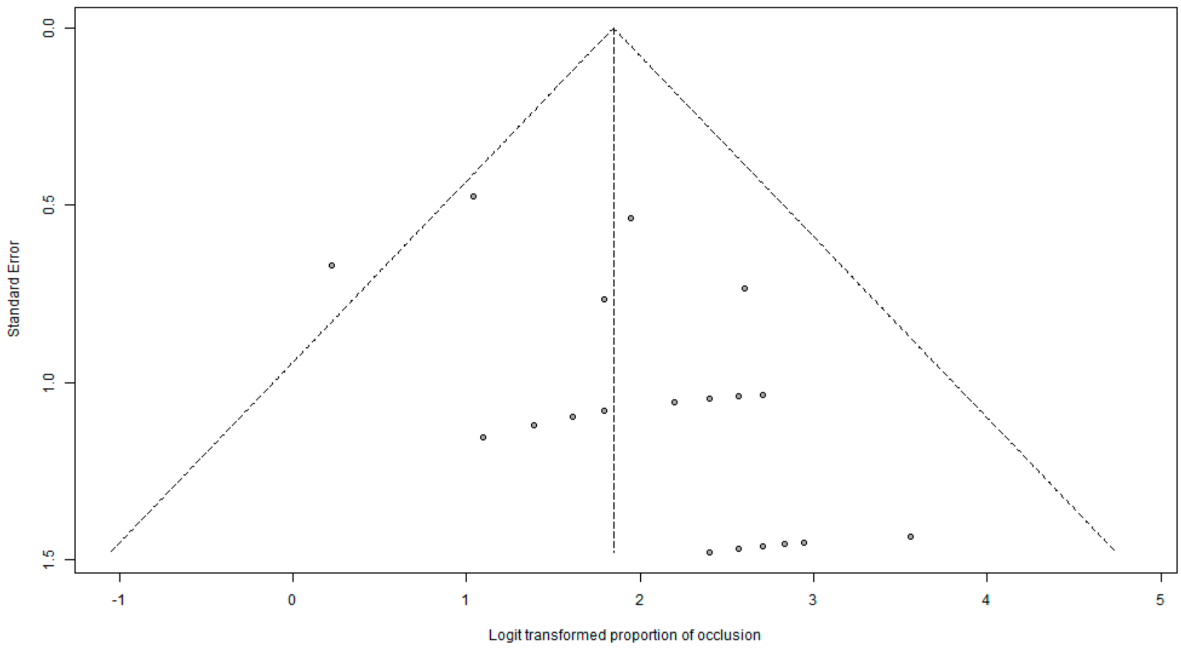
0.0
0.5
1.0
1.5
Standard Error
-1
0
1
2
3
4
5
Logit transformed proportion of occlusion

Table S1: Study characteristics and baseline characteristics per study

Study name	Design	N partici-pating centers	Inclusion period (mm-yyyy)	Total N of patients in study	N of eligible patients // aneurysms	Female sex (%)	Unfavorable HH (4-5)/ WFNS (4-5) (N, %)	An. Anterior circulation (N, %)	An. type	An. mean size (mm)	Mean treatment delay (days)
Aguilar-Perez 2020	R	1	UN	8	7//7	43	5 (71)	3 (43)	5xDIS<br>1xBBL<br>1xMYC	UN	3
Aydin 2015	R	3	01-2009 – 01-2013	11	11//11	82	1 (9)	9 (82)	11x BBL	3	10
Baker 2020	R	11	UN	23	23//23	61	8 (35)	0 (0)	8xDIS<br>7xSAC<br>4xBBL<br>3xPA<br>1xFUS	7	All within 1 week of diagnosis
Bhogal 2018	R	1	02 – 2009 – 02– 2016	7	7//7	57	1 (14)	5 (71)	7xSAC	3	6
Ten Brinck 2019	R	6	03-2012 – 12-2017	44	44//44	64	14 (32)	24 (55)	13x FUS<br>11x SAC<br>11x DIS<br>9xBBL	9	3

Cerejo 2017	R	1	06-2011 – 06-2016	8	7//7	71	2 (29)	7 (100)	7xBBL	3	6
Chalouhi 2015	R	2	01-2012 – 01-2014	20	16//16	88	0 (0)	11 (69)	12x SAC<br>4xDIS	7	4
Chan 2014	R	1	12-2010 – 02-2013	8	8//8	63	2 (25)	0 (0)	8xDIS	3	3
Da Ros 2020	R	2	01 – 2013 – 07 - 2019	7	6//6	UN	0 (0)	0 (0)	6xSAC	2	5
Ghorbani 2019	R	1	07 – 2014 – 03–<br>2016	18	17//17	59	0 (0)	14 (82)	12x SAC<br>5xBBL	2	4
Goertz 2018	R	3	02-2016 – 03-2018	10	10//11	70	3 (30)	9 (82)	4xSAC<br>3xBBL<br>3xDIS<br>1xFUS	4	All within 24h after hospital admis-sion[1]
Guerrero 2018	R	1	11 – 2014 – 04– 2017	9	9//9	33	4 (44)	0 (0)	9xDIS	5	1
Kaschner 2019	R	1	2016 –2018	10	10//11	50	4 (40)	5 (50)	7xDIS<br>3xBBL	8	1
Lin 2015	R	5	2011 –2013	26	18[3]//18	UN	6 (33)	15 (83)	8xBBL<br>6xDIS<br>2xSAC<br>2xFUS	7	UN

Linfante 2017	R	2	11-2013 – 11-2015	10	10[3]//10	UN	1 (10)	10 (100)	10x BBL	2	UN
Lozupone 2018	R	1	01-2009 – 02-2005	17	16//16	69	6 (38)	10 (63)	9xDIS<br>7xBBL	5	4
Mahajan 2018	R	1	06-2016 – 03-2018	16	15//15	UN	1 (7)	13 (87)	9[2]x SAC<br>1xFUS<br>5xBBL	4	5
Manning 2019	R	3	07 – 2015 – 09– 2018	14	14//14	86	6 (43)	8 (57)	7xSAC<br>5xFUS<br>2xBBL	9	UN
Maus 2018	R	3	11-2011 – 11-2017	15	14//14	36	9 (64)	0 (0)	14x DIS	6	All within 12h after hospital admis-sion[1]
McAuliffe 2012	R	3	08-2009 – 08-2010	11	6//6	67	2 (33)	3 (50)	2xSAC<br>2xFUS<br>2xBBL	17	3
Mokin 2018	R	14	11-2011 – 04-2017	43	43[3]//45	67	14 (33)	45 (100)	45x BBL	2	UN
Natarajan 2017	R	1	06-2011 – 06-2016	11	11[3]//14	73	4 (36)	11 (79)	5xSAC<br>1xFUS<br>6xBBL	5	3

									2xDIS		
Parthasarathy 2018	R	1	05-2014 – 07-2015	9	7//7	57	0 (0)	7 (100)	7xBBL	2	5
Ryan 2017	R	1	10-2013 – 11-2016	13	13//16	85	2 (15)	16 (100)	16xBBL	2	3
Wallace 2019	R	4	UN	35	6//6	83	1 (17)	0 (0)	3xDIS 2xFUS 1xSAC	6	All within 48 hours after admission
Yang 2017	R	1	06-2010 – 01-2017	13	9//10	67	UN	10 (100)	10x BBL	4	6

An . = aneurysm; BBL = blood blister-like; DIS = dissecting; FUS = fusiform; HH = Hunt and Hess; MYC = mycotic; N = number; P = prospective observational study; PA = pseudoaneurysm; R = retrospective analysis; SAC = saccular; UN = Unknown/Unclear; WFNS = World Federation of Neurosurgical Societies

[1]: Corresponding authors of both articles were mailed and confirmed all patients were treated within 15 days after last moment of hemorrhage.

[2]: Four of these aneurysms were described as ‘fusisaccular’.

[3]: Duplicate patients have been excluded.

Table S2: Types and number of used flow diverters

**Study**	Type of FD used (N patients)	Number of stents used
Aguilar-Perez 2020	P48 HPC (7)	6x 1 FD<br>1x 5 FDs
Aydin 2015	Silk (11)	8x 1 FD (1 + stent)<br>3x 2 FDs
Baker 2020	PED (23)	20x 1 FD (5 + coiling)<br>3x 2 FDs (1 + coiling)
Bhogal 2018	P64 (7)	6x 1 FD<br>1x 2 FDs
Brinck ten 2019	Derivo (3)<br>PED (4)<br>SILK (4)<br>FRED (12)<br>Surpass (21)	33x 1 FD (1 + stent, 7 + coiling)<br>8x 2 FDs (2 + coiling)<br>3x 3 FDs
Cerejo 2017	PED / PED flex (7)	1x 1 FD<br>6x 2 FDs
Chalouhi 2015	PED (16)	Not specified for included subgroup<br>Out of 20 patients in total study: 1x FD in 19/20.
Chan 2014	PED (8)	Mean number of FDs per patient is 1.4. Not further specified. Two patients received FD + coiling.
Da Ros 2020	P48 (1)<br>PED Shield (1)<br>PED (4)	5x 1 FD<br>1x 2 FDs
Ghorbani 2019	SILK Plus (3)<br>Surpass streamline (4)<br>PED (10)	Not specified
Goertz 2018	Derivo (10)	10x 1 FD (2 + coiling)
Guerrero 2018	PED (9)	8x 1 FD<br>1x 2 FDs
Kaschner 2019	Derivo (10)	9x 1 FD<br>1x 2 FDs
Lin 2015	PED (18)	12x 1 FD (3 + coiling)<br>5x 2 FDs (2 + coiling)<br>1x 4 FDs
Linfante 2017	PED (10)	9x 1 FD<br>1x 3 FDs
Lozupone 2018	SILK (1)<br>Surpass (1)<br>PED FLEX (2)<br>PED (12)	12x 1 FD (1 + coiling)<br>4x 2 FDs
Manning 2019	PED Shield (14)	Mean: 1.2 FD per patiënt, not further specified (12 + coiling)
Mahajan 2018	Surpass (15)	15x 1 FD (3 + coiling)
Maus 2018	FRED (3)<br>PED (11)	9x 1 FD (1 + stent)<br>2x 2 FDs (1 + coiling)<br>3x 3 FDs (3 + coiling)
McAuliffe 2012	PED (6)	3x 1 FD (3 + coiling<br>2x 2 FDs (1 + coiling)<br>1x 3 FDs
Mokin 2018	PED (43)	41x 1 FD<br>2x 2 FDs<br>(coiling unknown)
Natarajan 2017	PED (11)	11x 1 FD (+ 1 coiling)
Parthasarathy 2018	PED (7)	7x 1 FD

Ryan 2017	PED (13)	11x 1 FD<br>2x 2 FDs
Wallace 2019	PED (4)<br>PED Flex (2)	6x 1 FD (+ 2 coiling)
Yang 2017	PED (9)	8x 1 FD (+ 4 coiling)<br>1x 2 FDs
	Total 357:<br>• P64 (7)<br>• P48 (8; of which 7 p48 with Hydrophilic Polymer Coating (HPC))<br>• FRED (15)<br>• SILK (19; of which 3 Silk Plus)<br>• Derivo (23)<br>• Surpass (41; of which 4 Surpass streamline)<br>• Pipeline Embolization Device - PED (237; of which 4 PED Flex and 15 PED Shield)<br>• PED/PED Flex (7)	FD = Flow diverter

Table S3: Outcomes per study

Study name	Complete occlusion (n/n, %)	Scale occlusion	Median/Mean angiographic FU time (months)	Favorable clinical outcome (n, %)	All-cause mortality (n/n, [%])	Median/Mean Clinical FU time (months)	Rebleeding (n, %)
Aguilar-Perez 2020	4/5 (80)	RR	6	2 (29)	2/7 (29)	6	0 (0)
Aydin 2015	9/9 (100)	RR	6	10 (91)	1/11 (9)	UN	0 (0)
Baker 2020	17/23 (74)	NA	UN	16 (70)	5 /23 (22)	15	0 (0)
Bhogal 2018	5/5 (100)	RR	3	6 (86)	0 (0)	UN	0 (0)
Ten Brinck 2019	27/29 (93)	RR	16	20 (46)	8/44 (18)	7.6 (mean)	5 (11)
Cerejo 2017	6/7 (86)	NA	8	6 (86)	0/7 (0)	12	0 (0)
Chalouhi 2015	11/12 (92)	NA	5	15 (94)	1/16 (6)	5	0 (0)

Chan 2014	8/8 (100)	NA	5	5 (63)	0/8 (0)	12	0 (0)
Da Ros 2020	5/6 (83)	NA	2	5 (83)	0/6 (0)	33	0 (0)
Ghorbani 2019	17/17 (100)	NA	6	17 (100)	0/17 (0)	6	0 (0)
Goertz 2018	9/10 (90)	OKM	8	10 (100)	0/10 (0)	8	0 (0)
Guerrero 2018	5/5 (100)	NA	UN	7 (78)	1/9 (11)	4	0 (0)
Kaschner 2019	6/6 (100)	OKM	6	6 (60)	1/10 (10)	11	0 (0)
Lin 2015	15/16 (94)	NA	UN	14 (78)	3/18 (17)	UN	1 (6)
Linfante 2017	9/9 (100)	RR	15	9 (90)	1/10 (10)	3	0 (0)
Lozupone 2018	12/14 (86)	NA	6-12 months	11 (69)	2/16 (13)	6-12 months	0 (0)
Mahajan 2018	13/14 (93)	OKM	3-6 months	14 (93)	1/15 (7)	3-6 months	0 (0)
Manning 2019	12/14 (86)*	RR	0 (7 days)	UN	3/14 (21)	0 (7 days)	2 (14)
Maus 2018	6/6 (100)	OKM	7	3 (21)	7/14 (50)	7	0 (0)
McAuliffe 2012	3/4 (75)	NA	6	3 (50)	2/6 (33)	Up to 6 months	2[1] (33)
Mokin 2018	28/32 (88)	NA	4	26/38 (68)	8/43 (19)	3	1 (2)
Natarajan 2017	9/9 (100)	NA	24	9 (82)	2/11 (18)	23	0 (0)
Parthasarathy 2018	6/7 (86)	NA	6	6 (85)	0/7 (0)	UN	0 (0)
Ryan 2017	5/9 (56)	NA	UN	10 (77)	2/13 (15)	UN	0 (0)
Wallace 2019	3/5 (60)	NA	12	5 (83)	0 (0)	UN	0 (0)
Yang 2017	7/7 (100)	RR	6-9 months	8 (89)	0/9 (0)	22	0 (0)
* complete and near complete occlusion (Raymond-Roy grades 1 and 2)							

Table S4: Complications per study

Study name	Complication rate (n/n, %)	Type[1]			Intra-procedural	Post-procedural		Leading to permanent deficit in N patients (n/n, %).
		Ischemic	Hemorrhagic	Other		≤ 30 days	>30 days	
Aguilar-Perez 2020	5/7 (71)	2	0	6	4	4	0	UN
Aydin 2015	1/11 (9)	1	0	0	0	1	0	1/11 (9)
Baker 2020	5/23 (22)	4	2	0	0	UN	UN	UN
Bhogal 2018	0/7 (0)	0	0	0	0	0	0	0/7 (0)
Ten Brinck 2019	20/44 (45)	9	10	6	5	18	2	12/44 (27)
Cerejo 2017	3/7 (43)	2	1	0	0	3	0	2/7 (29)
Chalouhi 2015	1/16 (6)	0	1	0	1	0	0	1/16 (6)
Chan 2014	1/8 (13)	1	1	0	0	2	0	UN
Da Ros 2020	1/6 (17)	1	0	0	0	1	0	1/6 (17)
Ghorbani 2019	0/17 (0)	0	0	0	0	0	0	0/17 (0)
Goertz 2018	2/10 (20)	1	0	1	1	1	0	1/10 (10)
Guerrero 2018	3/9 (33)	0	3	0	0	1	0	1/9 (11)
Kaschner 2019	5/10 (50%)	1	3	2	1	5	0	UN
Lin 2015	5/18 (28)	3	2	0	2	3	0	2/18 (11)
Linfante 2017	0/10 (0)	0	0	0	0	0	0	0/10 (0)
Lozupone 2018	4/16 (25)	1	2	1	2	2	0	2/16 (13)
Mahajan 2018	1/15 (7)	1	0	0	1	0	0	0/15 (0)
Manning 2019	5/14 (36)	3	2	0	0	5	0	1/14 (7)

Maus 2018	4/14 (29)	3	1	0	4	1	0	1/14 (7)
McAuliffe 2012	3/6 (50)	2	3	0	2	2	0	2/6 (33)
Mokin 2018	9/43 (21)	5	3	1	7	1	1	UN
Natarajan 2017	2/11 (18)	2	1	0	2	1	0	1/11 (9)
Parthasarathy 2018	0/7 (0)	0	0	0	0	0	0	0/7 (0)
Ryan 2017	5/13 (39)	1	3	1	2	3	0	3/13 (23)
Wallace 2019	1/6 (17)	0	0	1	1	0	0	0/6 (0)
Yang 2017	1/9 (11)	0	1	0	0	0	1	1/9 (11)
Total pooled	87/357 (24)	43	39	19	35	54	4	32/265 (12)
[1]:The total amount of complications presented per subtype may exceed the number mentioned in the complication rate. This is due to the fact that some patients had multiple complications.								

Table S5: Quality of evidence

Study name	Patient N	Precision	Study quality* (GRADE)
Aguilar-Perez 2020	7	Serious	⊕○○○ Very low
Aydin 2015	11	Serious	⊕○○○ Very low
Baker 2020	23	Serious	⊕○○○ Very low
Bhogal 2018	7	Serious	⊕○○○ Very low
Ten Brinck 2019	44	Serious	⊕⊕○○ Low

Cerejo 2017	7	Very serious	⊕○○○ Very low
Chalouhi 2015	16	Very serious	⊕○○○ Very low
Chan 2014	8	Very serious	⊕○○○ Very low
Da Ros 2020	6	Very serious	⊕○○○ Very low
Ghorbani 2019	17	Very serious	⊕○○○ Very low
Goertz 2018	10	Very serious	⊕○○○ Very low
Guerrero 2018	9	Very serious	⊕○○○ Very low
Kaschner 2019	10	Very serious	⊕○○○ Very low
Lin 2015	18	Very serious	⊕○○○ Very low
Linfante 2017	10	Very serious	⊕○○○ Very low
Lozupone 2018	16	Very serious	⊕○○○ Very low
Mahajan 2018	15	Very serious	⊕○○○ Very low
Manning 2019	14	Very serious	⊕○○○ Very low
Maus 2018	14	Very serious	⊕○○○ Very low
McAuliffe 2012	6	Very serious	⊕○○○ Very low
Mokin 2018	43	Serious	⊕⊕○○ Low
Natarajan 2017	11	Very serious	⊕○○○ Very low
Parthasarathy 2018	7	Very serious	⊕○○○ Very low
Ryan 2017	13	Very serious	⊕○○○ Very Low
Wallace 2019	6	Very serious	⊕○○○ Very Low
Yang 2017	9	Very serious	⊕○○○ Very low
* Observational studies start at ‘low’. Due to already solely the issues regarding the precision (amongst others number of participants or events) all these studies must be judged to be of (very) low quality. We did not provide overview of other points of assessment (directness, publication bias) since this would also only yield serious/very serious issues and not change the final outcome of (very) low quality.			
